# High side-mode suppression ratio with a Vernier effect single-mode laser using triple coupled microrings

**DOI:** 10.1038/s41598-023-34267-9

**Published:** 2023-05-01

**Authors:** Milad Jahangiri, Fatemeh Moradiani, Gholam-Mohammad Parsanasab, Mohsen Mirmohammadi

**Affiliations:** Integrated Photonics Laboratory, Faculty of Electrical Engineering, Shahid Beheshti University, Tehran, Iran

**Keywords:** Optics and photonics, Lasers, LEDs and light sources, Optical materials and structures

## Abstract

The development of single-mode lasers with a high side-mode suppression ratio (SMSR) is challenging but highly desirable for integrated photonics devices and long-distance communications due to their high spectral purity and stability. Here, we demonstrate a single-mode laser with a high side-mode suppression ratio based on size-mismatched triple-coupled microrings. With the exact engineering of several key parameters of the structure like air gap and radii of microrings for controlling the free spectral range (FSR), a predominant mode is selected to lase in amplified spontaneous emission (ASE) of the gain material and all side and high order modes are suppressed by Vernier effect. In this work, we show that a single-mode operation is efficiently generated with an improved side-mode suppression ratio of over 20 dB in a three-ring-coupled microcavity laser. The single-frequency output persists for a wide power range. The theoretical calculations and numerical simulations’ results confirm the validity of the experimental results. Our structural engineering creates new opportunities in a variety of frontier applications in single-mode lasers and high-quality sensors.

## Introduction

Wide applications of microcavities, especially microrings based on organic materials, in the fields of bio-sensors and telecommunications have made this issue one of the prominent topics in the world of optical systems in recent years^1–3^. These structures not only have wide applications but also a fast, simple, and inexpensive fabrication process. Despite the widespread use of these types of microring lasers, due to the lack of mode selection capability, particularly when using a single ring, the single-mode operation with a high side-mode suppression ratio is challenging to achieve. There are several methods to obtain a single-mode operation and remove most of the longitudinal and side modes. Many approaches have been put out to address this issue, including the Vernier effect^4–6^, loss engineering^7,8^, and parity-time symmetry structures^9^. The most important of these methods is structural engineering based on the Vernier effect^10,11^. In this paper, we first demonstrate several exciting modes were eliminated based on the Vernier effect in asymmetric double microrings structure. Nevertheless, some of the high order and side modes still remained in the output spectrum, which is challenging for special applications such as highly sensitive sensors, optical networks, and long-distance communications^12,13^. To completely remove all these side and high order modes, which is the main goal of this paper, we employed microcavity structure engineering and presented a new structure of size-mismatched triple mutually microrings laser. We were able to successfully remove all these side modes and achieve a pure single-mode lasing for a wide power range. In some previous research based on multi microrings or disks structures, there are still side or high-order modes in high pump powers. Because some of the main and side modes in the material's gain area are activated in high pumps and stimulated under mode competition, therefore the operation of single-mode lasing is endangered in higher pumps^14–17^. To address this challenge, we need to use a different structure compared to the previous works so that by utilizing the design of adjustable parameters in the fabricating process, such as the radius of the microrings and the air gap in the size-mismatched triple mutually microrings laser, this single-mode operation can still be maintained in high pumps without any side or high order modes. In the previous works, all the structures presented in the arrangement of three, multi or cascading rings are either passive or have been investigated theoretically and numerically for various applications such as filters and creating a delay line in signal processing by methods of delay line signal processing and transfer matrix theory^11,18–21^.

To better understand this issue, our purpose is to design a side-mode suppressed multi-coupled microrings laser experimentally to eliminate all the side, bonding, and anti-bonding modes according to the Vernier effect and yield an extremely sharp single-mode lasing with high side-mode suppression ratio (SMSR) output^22–26^. We demonstrate that the side-mode suppression ability of coupled microring lasers can be improved by adding one more coupling size mismatched microring to form the cavity. FSR of eigenmodes is inversely proportional to the size of the resonator. To achieve a large FSR, a cavity with a radius of several microns is often required, which inevitably increases the optical loss, deteriorates the laser *Q* factor, and ultimately leads to an increase in the pump threshold. An effective alternative is to use multiple resonators coupled with each other with different radii to improve the FSR of the system via the Vernier effect^27–29^. Based on the Vernier effect, two microrings with a radius difference of 1µm have been chosen thanks to structural engineering. This small radius difference increases the free spectral range (FSR) which can select only one of the eigenmodes in the ASE of the gain material and lead to a single-mode operation^4,27^. In comparison to the other two microrings, the other microring has a radial difference in the order of 4 and 5 µm. The FSR is decreased as a result of the rise in the radius difference, which also effectively reduces the amplitude of the side modes and can help to remove all these side modes gradually. Therefore, a pure single-mode spectrum with high SMSR is produced by combining these two structures. In addition to single-mode lasing, this structure is more stable compared to other structures. It should be explained here that when the radius difference in two microrings is high, it is possible that the side modes may not exist in the output spectrum, however, because the FSR is small, several exciting modes may exist in the amplified spontaneous emission (ASE) of the material and the single-mode lasing may not be obtained. Here, we theoretically investigate a comprehensive analysis of a laser system consisting of three coupled microrings.

## Theoretical analysis

**Figure 1:**
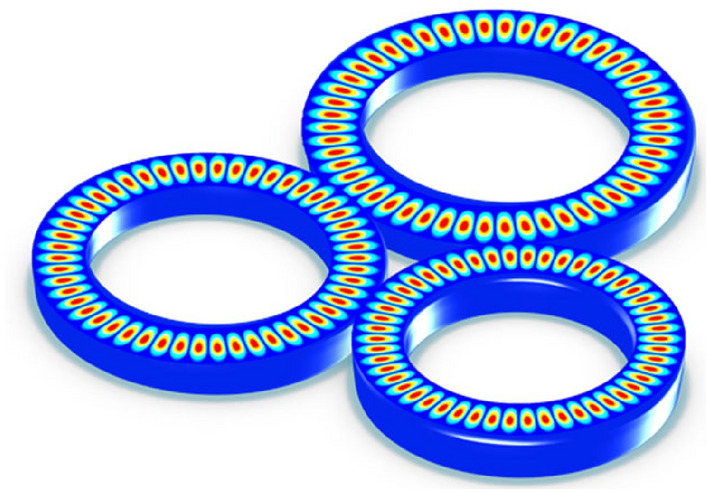
Schematic of field distribution in a triple coupled microrings laser where all modes have been coupled as a dominant mode.

A significant field of research is taking shape around the control of coupling between optical waveguides and microresonators. We have investigated the laser configuration shown in Fig. 1 consisting of optical microcavities formed by three coupled microrings with radii of R_1_ = 30 µm, R_2_ = 34 µm, and R_3_ = 35 µm. We characterize the interaction by the matrix relation when just one unidirectional mode of the resonator is activated and the coupling is lossless. The bidirectional interaction between two coupled microrings is shown theoretically in^15^. We employ Eq. (1) to conceptually investigate the lasing performance of a triple microring laser.

**Figure 2:**
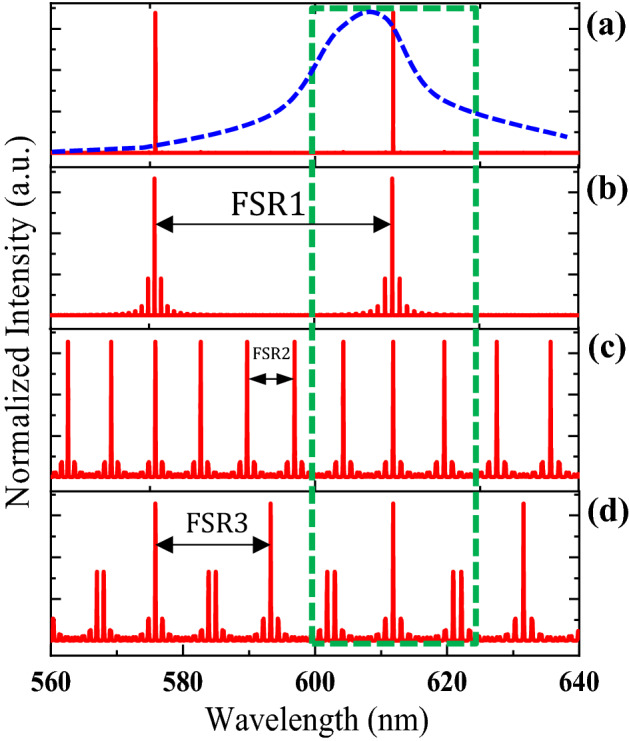
Normalized theoretical spectra of (**a**) triple microrings laser with radii of ${\mathrm{R}}_{1}$, ${\mathrm{ R}}_{2}$, ${\mathrm{ R}}_{3}$ = 30, 34, 35 µm (solid red line), ASE spectrum (dashed blue line) and double microring resonators with radii of (**b**) ${\mathrm{R}}_{1} , {\mathrm{R}}_{2}$ = 35, 34 µm, (**c**) ${\mathrm{R}}_{1}$, ${\mathrm{ R}}_{3} =$ 30, 35 µm, (**d**) ${\mathrm{R}}_{2}$, ${\mathrm{ R}}_{3}$ = 30, 34 µm. The green dotted box indicates that all of the microring resonators have the same resonance wavelength at 612 nm.

It shows the tri-directional relationship between the three coupled microrings.

1 $${\text{I}} = \prod\limits_{{\text{i = 1}}}^{3} {\left( {\frac{{e^{{ - 4\pi \alpha r_{{\text{i}}} }} \kappa^{2} }}{{1 + e^{{ - 4\pi \alpha r_{{\text{i}}} }} (1 - \kappa^{2} ) - 2e^{{ - 4\pi \alpha r_{{\text{i}}} }} \sqrt {1 - \kappa^{2} } \cos \left( {\frac{{4\pi^{2} n_{eff} r_{{\text{i}}} }}{\lambda }} \right)}}} \right)}$$

where α ∼ 10^(−7)^ represents the attenuation coefficient of the guided modes of the rings, n_eff_ = 1.558 is the effective refractive index of the whole of the structure, λ indicates the vacuum wavelength, and κ = 0.35 is the coupling coefficient. Regarding the choice of the coupling coefficient in the theoretical relationship, some experimental considerations have been taken into account. At this value of the coupling coefficient, the experimental curves are well-matched with the theory. At values greater than 0.35, the amplitude of the side modes increases and the SMSR decreases. To compare two, and triple-coupled microring lasers theoretically, the circulating power relation was calculated via MATLAB software. To compare all cases, the spectra are normalized and the results are displayed in Fig. 2. Microring lasers are potential candidates for in-plane integrated lasers; however, they exhibit multimode lasing spectra. Because of various optical losses in microrings, for achieving experimental output powers, the radius of the microring laser should be in the tens of microns range, therefore, the laser operates in the multimode lasing regime. Here, we theoretically propose and experimentally demonstrate a novel approach for achieving a pure single-mode lasing with a high side-mode suppression ratio. Our approach is based on using a triple-coupled microrings structure. In order to show a high SMSR, first different double microrings structures with the same material and conditions with radii of R_1_, R_2_ = 30, 34 µm, R_1_, R_3_ = 30, 35 µm, and R_2_, R_3_ = 34, 35 µm were fabricated and their output spectrums were obtained.

In addition to the theoretical analysis presented above, there are other approaches to express the number and the way of coupling microrings in one-dimensional (1D) and two-dimensional (2D) arrangements described in^30–33^. The synthesis of the transfer functions of parallel coupled ring resonators in 1D arrangement using a recursive algorithm is presented by Little et al. in^30^. A complex matrix formalism employing racetrack ring resonator filters is derived from Griffel^31^. A technique using simple closed-form formulas to determine the Q factor of each involved ring resonator which leads to the coupling coefficients is demonstrated by Melloni^32^. The analysis of the 2D arrangement of microrings using a transfer function is given in^33^ in detail. This approach has clearly shown that with the increase in the number of microrings, the quality coefficient of the output spectrum emitted increases, as a result, it is possible to increase the linewidth and SMSR by increasing the quality Factor. But the lasing threshold is highly dependent on the design of the structure such as the radius of fourth, fifth, or higher microrings and disposition of the microrings. In the triple microrings laser with the two-dimensional arrangement proposed in this paper, there are three simultaneous coupling regions (Fig. 1), which by choosing the right radius of the microrings, only one dominant mode is amplified, and the other modes are completely removed and the single-mode lasing is obtained.

All the various double microrings structures fabricated with specified dimensions according to the Vernier effect have many side or high order modes with low side-mode suppression ratio in agreement with the theoretical results in Fig. 2. In order to solve this problem and completely eliminate these side or high order modes, we proposed triple microrings structure based on the same dimensions and material in the double microrings structures. We were able to successfully eliminate all the side, bonding, and anti-bonding modes in the output spectrum of the triple microrings structure based on Eq. (1). This helps us to have a high SMSR single-mode on-chip laser source that can be used in very sensitive applications like optical communications, information processing, sensors, and optical filters^12^. The calculated spectra for the different double microrings are shown in Fig. 2. As can be observed, as the radius difference of the rings increases, the FSR reduces. Contrastingly, the amplitude of the side modes is comparatively greater in the case where the difference in the ring radii is smaller. In summary, the diagram of FSRs for a dominant mode selection is illustrated in Fig. 2. As Fig. 2 shows, the three-ring-coupled cavity has a larger difference in intensity between the main and side modes—as a result, the ratio between the threshold of the side and the main mode is higher. The simulated results qualitatively support the experimental findings.

**Figure 3:**
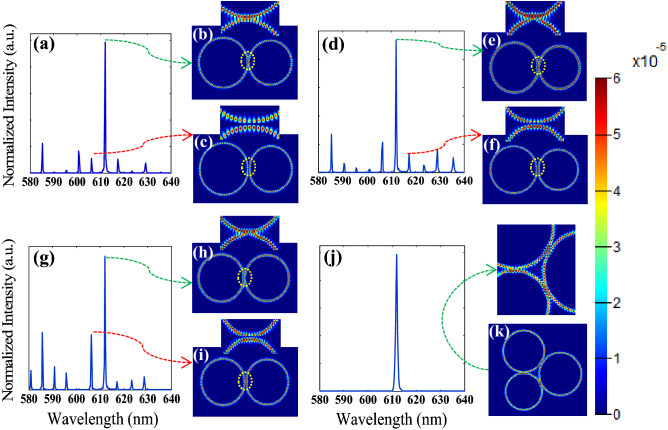
Normalized numerical simulation spectra of triple and double microring resonators with different radii (${\mathrm{R}}_{1}=3 \mu m$, ${\mathrm{ R}}_{2}=3.4\mu m$, ${\mathrm{R}}_{3}=3.5 \mathrm{\mu m}$) and electric field profile at the specified wavelengths, (**a**) ${\mathrm{R}}_{1}$, ${\mathrm{ R}}_{2},$ (**b**) electric field profile at 612 nm, (**c**) electric field profile at 606.2 nm, (**d**) ${\mathrm{R}}_{1}$, ${\mathrm{R}}_{3}$, (**e**) electric field profile at 612 nm, (**f**) electric field profile at 617.4 nm, (**g**) ${\mathrm{R}}_{2}$, ${\mathrm{R}}_{3}$, (**h**) electric field profile at 612 nm, (**i**) electric field profile at 606.2 nm, (**j**) ${{\mathrm{R}}_{1},\mathrm{ R}}_{2}$, ${\mathrm{ R}}_{3}$, (**k**) electric field profile at 612 nm, Insets: enlarged view of the coupling regions. The color bar shows the amount of coupling strength in different excited modes.

Therefore, using the combination of these two designs and setting up the triple coupled microrings configuration satisfies a single-mode lasing with high side-mode suppression ratio. The result is shown in Fig. 2a. In order to better demonstrate the removing of side modes by triple microrings laser, double microrings structures with smaller relative radius (R_1_ = 3, R_2_ = 3.4, and R_3_ = 3.5 µm) have been simulated with finite difference time domain (FDTD) method, and their output spectrum and electric field profile are illustrated at the specified wavelengths in Fig. 3a–k. As a result, many high order and side modes effectively exist in the output spectrum of double microrings structures and there is no side or high order modes in output spectrum of the triple microrings laser, therefore leading to a higher SMSR. If we look closely at the coupling region of the profile at the main mode, the constructive coupling has occurred in the structure, which strengthens the main mode. While this has not happened at the other side modes.

## Experimental process and results

In this section, an experimental construction on the triple microrings laser configuration is presented. To make the material of these structures, 8 mg of Rhodamine-B as a gain material is solved with 16 g of SU-8. After making the material, it is time to spin coat this material on a layer of silicon dioxide as a substrate that has been washed and cleaned before. We used a spin-coat machine at a speed of 4000 rpm, which creates a thickness of 2 µm. The thickness was measured by Filmetrics (F10-RTA) device, which has high accuracy. Then we pre-baked the layer for 2 min at 95 °C. To write the structure on the SU-8 layer, we used a direct-writing lithography method. The beam of 400 nm laser is focused on the Rhodamine-B doped SU-8 material by using a 50 × lens. Laser power should be adjusted according to the width of the microrings. To create a width of 1.5 µm, the writing power of the laser was set to 0.5 mw. Then the sample was post-baked at 95 °C for 8 min. When the sample has been exposed to the beam of laser, the desired pattern remains, and in the rest of the places, the material is removed by the solver.

**Figure 4:**
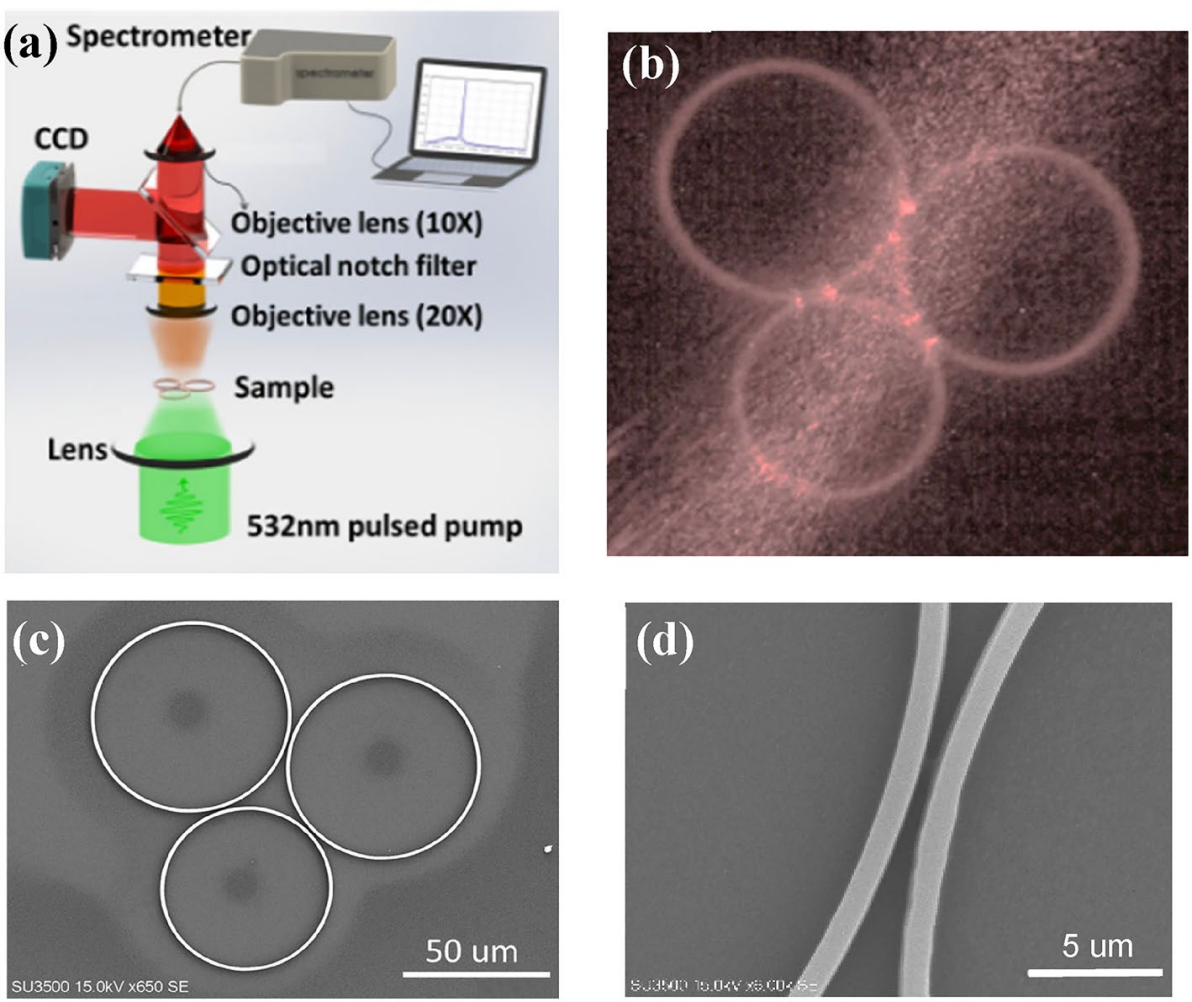
(**a**) Schematic of the experimental set-up used for laser emission characterization. (**b**) The CCD camera image of triple coupled microrings configuration and its pump by 532 nm pulsed laser. (**c**) Scanning electron-microscope (SEM) image of triple coupled microrings. (**d**) The microrings separated by 0.6 µm air gap.

This process is done by placing the sample inside the solver of SU-8 material for one minute. Finally, the sample is hard-baked for 2 h at a maximum temperature of 150 °C to increase the durability of the design on the substrate. Figure 4a shows the schematic of the experimental set-up used to characterize the laser spectrum, where the 532 nm pulsed pump laser passes through a polarizer to adjust and measure the power. The spectrum scattered from the designed structure is collected by a 20 × lens and passes through an optical notch filter to filter the pump light, then a 10 × lens focused on the sample transmits the emission to a fiber attached to the spectrometer. Figure 4b illustrates a CCD camera image of fabricated triple microrings coupled together and its pump by a 532 nm pulsed laser. The spot size of the pulsed pump laser is 0.3 mm^2^ which assures us that the whole of the structure is simultaneously pumped uniformly. The theoretical model was established using the Vernier effect. Figure 4c, d show a scanning electron microscope (SEM) image of a coupled triple microrings arrangement. In our current analysis, the rings’ dimensions (radii, widths, and heights) are selected so as to support a high-side mode suppressed lasing in the radial direction. Compared to the double coupled microring resonators, a narrower transmission spectrum and pure single-mode with no side and high order modes (high SMSR) have been achieved in our system which means better sensitivity and high resolution for laser spectroscopy and optical fiber communications application^26^.

In order to clearly examine all the advantages of the triple microrings structure presented in this paper, it is necessary to carefully determine the experimental and simulation spectra of the single, double, and triple microrings arrangements. For this purpose, in the first step, all the structures are simulated with the FDTD method, and their output spectrum is obtained. For high precision simulation, the gain is considered for all microrings. The boundary condition of perfect match layer (PML) is used to absorb the outgoing waves.

**Figure 5:**
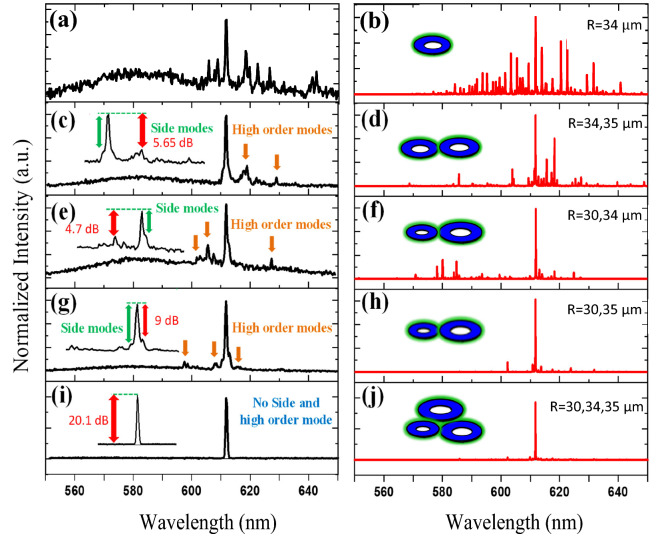
Comparison of single microring resonator spectra with radius R = 34 µm in terms of (**a**) experiment and (**b**) simulation, Comparison of double microring resonators spectra with radii of R = 34, 35 µm in terms of (**c**) experiment and (**d**) simulation, Comparison of double microring resonators spectra with radii of R = 30, 34 µm in terms of (**e**) experiment and (**f**) simulation, Comparison of double microring resonators spectra with radii of R = 30, 35 µm in terms of (**g**) experiment and (**h**) simulation, Comparison of triple microrings laser spectra with radii of R = 30, 34, 35 µm in terms of (**i**) experiment and (**j**) simulation. High SMSR lasing signal observed in triple microring laser configuration experimentally. The highest SMSRs are 5.65, 4.7, and 9 for the two-ring cavities structures and 20.1 dB for a three-ring cavity.

For the experimental results, the structures were fabricated with the same material on a substrate and their results were obtained with the spectrometer under the same conditions. Finally, the simulation and experimental results for the desired structures are illustrated in Fig. 5. To compare all cases, the spectra are normalized at the same pump energy (~  58.29 μJ/mm^2^) and the results are indicated in Fig. 5. As it can be seen clearly, the side and high order modes exist in the double microrings output spectrums that are marked with green and orange arrows, but in the triple microrings laser, in addition to a narrower linewidth, these side and high order modes have also effectively been removed. The experimental data shows a very excellent match with the simulation data. Figure 5c, e, g, i show the corresponding SMSR for various configurations of asymmetric double coupled microrings and triple microring laser. As a result, we could enhance the laser intensity with high SMSR in triple coupled microring laser over 20 dB which it is much higher than that of various two-ring-coupled cavities (9, 5.65, 4.7 dB). The ratio between the threshold of the side and the main mode (I_sth_/I_th_) is about 2 in three-ring-coupled cavity.

**Figure 6:**
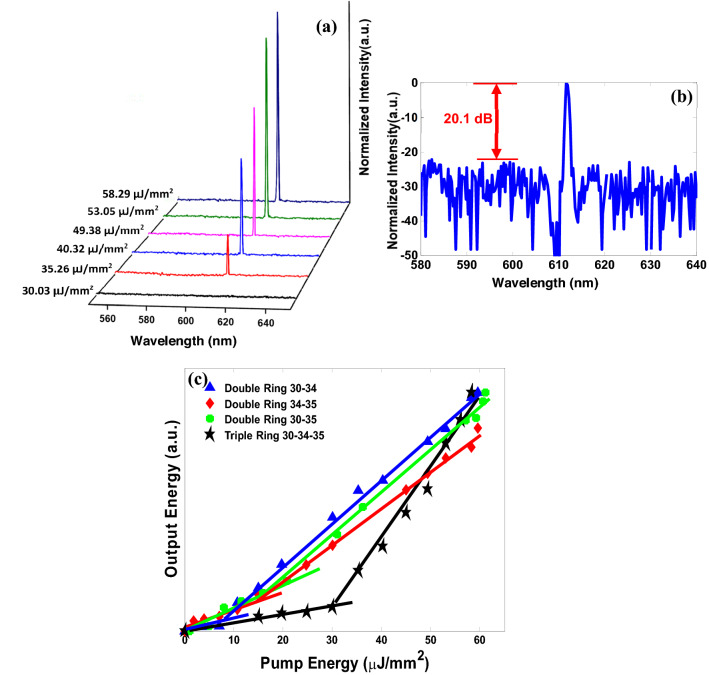
(**a**) Single-mode lasing spectrum of triple microrings laser with radii of 30, 34, and 35 µm for different pump energies, the exciting laser wavelength is achieved at 612 nm with 0.6 nm FWHM (spectrometer resolution is 0.5 nm) and is highly stable with increasing pump energy. (**b**) Improved corresponding SMSR for triple mutual microrings laser that is more than 20 dB. (**c**) The normalized output emission intensity of triple microrings laser compared to double microring resonators as a function of pump energy. The solid black line shows the triple microrings lasing threshold of∼31 µJ/mm^2^. A high SMSR lasing signal was observed at a pump power of ~ 58.29 μJ/mm^2^.

When the sample is pumped with a 532 nm pulsed laser, the triple microrings laser has a single-mode lasing emission at the wavelength of 612 nm with 0.6 nm Full width at half maximum (FWHM), while this value for different double microrings configurations is 0.8 nm. Figure 6a shows the triple microrings laser spectrum in different pump powers. The stability of the laser is very suitable for pump powers much higher than the threshold pump energy. According to the FSR formula^13,27^, there is only one exciting mode in the ASE spectrum (wavelengths from 580 to 660 nm) shown in Fig. 5i. As can be seen in Fig. 6a, the triple microrings laser output spectrum has been significantly improved with high SMSR, and all high order or side modes have been eliminated by engineering the structure of triple coupled microrings, also a pure single-mode emission for different pump energies has been achieved experimentally, which is theoretically demonstrated in Fig. 2a and simulated shown in Fig. 5j. To clearly show the SMSR for three coupled microrings arrangements Fig. 6b is presented. Figure 6b indicates the normalized logarithmic spectrum of triple microrings laser which represent an improvement of corresponding SMSR for this configuration over 20 dB in comparison with double microrings structures. The highest SMSRs are 5.65, 4.7, and 9 for the two-ring cavities structures. Figure 6c represents the normalized output emission intensity of the integrated triple microrings laser compared to the double microring resonators as a function of pumped energy. The lasing thresholds of various double microring structures are about 16, 17, and 8 µJ/mm^2^. The solid black line in Fig. 6c shows the lasing threshold of triple coupled microrings laser, which is ∼ 31 µJ/mm^2^. We experimentally measured the scattered emitted light power from the microring laser, which is in the order of 50–500 nW. The emitted light power is dependent on the fabrication process, geometrical structure, and measuring set-up arrangement. It is expected with increasing the pump power rate, the laser can maintain its stability, and definitely, a laser with a higher SMSR can be achieved.

## Conclusion

In conclusion, we have especially investigated a Vernier effect triple microrings laser with a high side-mode suppression ratio based on Rhodamine-B doped SU-8 as an organic active material which provides a suitable context for fabricating multi-microring structures. The main configuration, which has three microrings coupled together is presented in this paper so that in addition to maintaining the stability of the single-mode operation, it is able to eliminate effectively all the side and high order modes based on the Vernier effect and generate a high side-mode suppression ratio (SMSR) that were proposed as the main goal. The SMSR of the single-mode laser reaches more than 20 dB. Our work provides an effective roadmap for designing on-chip light sources. Due to its simplicity in construction, low cost, and its high sensitivity, it has the ability to be effectively used in optical integrated circuits for wide applications like optical sources, long-distance communications systems, biomedical sensors, and different types of optical gyroscopes.

## Data Availability

The datasets generated and analyzed during the current study are available from the corresponding author on reasonable request. This is to certify that all authors have seen and approved the manuscript being submitted & have no conflict of interest. We would like to submit the paper entitled “High Side-Mode Suppression Ratio with a Vernier effect Single-mode Laser Using Triple Coupled Microrings” for possible evaluation in the Journal of Scientific Reports. We affirm that the manuscript has been prepared according to the Journal’s instructions and the content of the manuscript has not been published in any refereed journal. I deeply appreciate it if I can meet the valuable referees’ comments and your decision.
